# Brain‐Derived Neurotrophic Factor (BDNF) as a Potential Biomarker in Brain Glioma: A Systematic Review and Meta‐Analysis

**DOI:** 10.1002/brb3.70266

**Published:** 2025-01-09

**Authors:** Fatemeh Hasani, Mahdi Masrour, Sina Khamaki, Kimia Jazi, Erfan Ghoodjani, Antonio L. Teixeira

**Affiliations:** ^1^ Gastroenterology and Hepatology Research Center Golestan University of Medical Sciences Gorgan Iran; ^2^ Neuroscience Research Center Golestan University of Medical Sciences Gorgan Iran; ^3^ School of Medicine Tehran University of Medical Sciences Tehran Iran; ^4^ Student Research Committee, Faculty of Medicine Medical University of Qom Qom Iran; ^5^ School of Medicine Isfahan University of Medical Sciences Isfahan Iran; ^6^ Biggs Institute The University of Texas Health Science Center at San Antonio San Antonio Texas USA

**Keywords:** BDNF, biomarker, brain neoplasms, brain‐derived neurotrophic factor, glioblastoma, glioma

## Abstract

**Background:**

This systematic review and meta‐analysis evaluates peripheral and CNS BDNF levels in glioma patients.

**Methods:**

Following PRISMA guidelines, we systematically searched databases for studies measuring BDNF in glioma patients and controls. After screening and data extraction, we conducted quality assessment, meta‐analysis, and meta‐regression.

**Results:**

Eight studies were included. Meta‐analysis showed significantly reduced plasma BDNF levels in glioma patients versus controls (SMD: −1.0026; 95% CI: [−1.5284, −0.4769], *p* = 0.0002). High‐grade gliomas had lower plasma BDNF (*p* = 0.0288). Tissue BDNF levels were higher in glioma patients (SMD: 1.9513; 95% CI: [0.7365, 3.1661], *p* = 0.0016) and correlated with tumor grade (*p* = 0.0122). Plasma BDNF levels negatively correlated with patient age (*p* = 0.0244) and positively with female percentage (*p* = 0.0007).

**Conclusion:**

BDNF is a promising biomarker in glioma, showing significant changes in plasma and tissue levels correlating with tumor grade, patient age, and gender.

## Graphical abstract

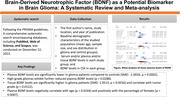

## 1 Introduction

Brain and central nervous system (CNS) cancer poses a significant global public health concern due to its high mortality and poor prognosis (Sung et al. 2021; Siegel et al. 2021). Gliomas are common primary brain malignancies affecting both pediatric and adult populations (Ostrom et al. 2016). As per the 2020 Global Cancer Observatory (GLOBOCAN) estimates, brain and CNS tumors hold the 19th position among the most frequent malignancies and the 12th leading cause of cancer‐related deaths (Sung et al. 2021). The overall prognosis associated with glioma is unfavorable, with a 1‐year survival rate of around 35.7% (Dolecek et al. 2012). Consequently, prompt diagnosis and therapeutic interventions are imperative to prolong life expectancy and/or enhance the quality of life. Numerous studies have explored the identification of a readily accessible marker for glioma (Ronvaux et al. 2022).

Brain‐derived neurotrophic factor (BDNF), a member of the neurotrophin family, plays a pivotal role in neuron development, growth, survival, and synaptic plasticity (Park and Poo 2013; Diniz et al. 2015; Nijs et al. 2015; Smith 2014). BDNF exerts its effects through tropomyosin receptor kinase B (TrkB), and its presence in gliomas may enhance tumor growth and contribute to the progression toward malignancy (Haapasalo et al. 1999; Ehsanifard et al. 2023). An increasing body of in vitro research indicates high BDNF expression in gliomas (Xiong et al. 2015; Xiong, Zhou, Lim, et al. 2013; Wang, Liu, and Song 2018). Tissue BDNF levels in glioma patients markedly exceeded those of normal controls, with higher BDNF levels corresponding to higher pathological grades (Yan, Yu, and Li 2009). BDNF expression has also exhibited a marked increase in high‐grade glioma tissues, displaying a positive correlation with both tumor malignancy and the expression of the TrkB receptor (Xiong et al. 2015). Similarly, BDNF mRNA levels have been reported to be higher in glioma tissues than in normal brain tissues (Wang, Liu, and Song 2018).

Due to the substantial worldwide health concern posed by gliomas, there is a pressing need for efficient diagnostic and therapeutic approaches. Multiple studies have demonstrated the importance of BDNF in gliomas and its association with the aggressiveness of the tumor. Nevertheless, these studies exhibit differences in their methodology, approaches, participant groups, and results, which may result in incongruities and ambiguities. Hence, there is a need for stronger and more dependable conclusions, which may direct future research directions and potentially guide clinical practice. This systematic review and meta‐analysis aim to investigate the potential of serum and tissue levels of BDNF as a biomarker in glioma.

## 2 Materials and Methods

### 2.1 Search Strategy

Our systematic review was conducted following the Preferred Reporting Items for Systematic Reviews and Meta‐Analyses (PRISMA) guidelines (Table S1) (Moher et al. 2009). A comprehensive systematic search encompassing international online databases, including PubMed, Web of Science, and Scopus, was conducted on December 22, 2023. The search utilized keywords such as “brain‐derived neurotrophic factor” AND “glioma,” along with other pertinent terms, which are available in detail in Table S2. The search was conducted without applying filters or limitations. The protocol for our review has been registered in PROSPERO under the registration number CRD42024499623.

### 2.2 Inclusion and Exclusion Criteria

Our review adhered to specific inclusion criteria: (1) studies reporting BDNF levels in glioma and controls within the serum, plasma, cerebrospinal fluid (CSF), or tissue; and exclusion of (1) studies not reporting BDNF levels (with attempts to contact corresponding authors for data and exclusion if data could not be obtained); (2) reviews, conference abstracts, and case reports; and (3) studies not published in English.

### 2.3 Screening

Following the elimination of duplicates from the initial search, two reviewers (F.H. and S.K.) independently reviewed titles and abstracts to identify relevant studies. Subsequently, the full texts of selected studies were evaluated for inclusion, with any disagreements resolved through discussion with a third reviewer (M.M.).

### 2.4 Data Extraction

Utilizing a predefined sheet, two authors (F.H. and M.M.) independently extracted data from the studies, encompassing (1) the first author's name, study location, and year of publication; (2) baseline demographic characteristics of the studied population (mean age, sample size, and sex distribution in glioma and control groups); (3) serum and/or plasma and/or tissue BDNF levels in each study group; and (4) BDNF levels in CSF in each group.

A few studies skipped reporting certain values required for us to perform meta‐analysis but presented the data in their graphical representations. The relevant data from these plots has been extracted using WebPlotDigitizer (Rohatgi 2020). In instances where data regarding BDNF levels were not explicitly provided, attempts were made to contact the corresponding authors.

### 2.5 Quality Assessment

The quality assessment of the included studies was conducted using the “Newcastle‐Ottawa Quality Assessment Scale” (NOS) for observational studies (Wells et al. 2000). The assessment was independently carried out by two authors (F.H. and S.K.), and any discrepancies were resolved through examination by a third reviewer (K.J.). The evaluation encompassed three primary bias categories: selection, comparability, and outcome. Scores falling within the ranges of 9–10, 7–8, 5–6, and less than 5 were categorized as “very good,” “good,” “satisfactory,” and “unsatisfactory,” respectively.

### 2.6 Statistical Analysis

The statistical analyses and visualizations were performed using R version 4.2.2 (R Core Team [2021], Vienna, Austria) with the help of the “meta” package (Balduzzi, Rücker, and Schwarzer 2019). We used the bias‐corrected Hedges' *g* standardized mean difference (SMD) to compare the mean BDNF levels in control groups and glioma patients. We then conducted a meta‐analysis on the obtained SMDs using the random effects model. Hedges' *g* was chosen because it reflects both the test and control groups' sample sizes when calculating the effect size (Hedges 1981). A meta‐analysis was also conducted using the random effects model to pool data on the mean BDNF level in glioma patients.

The random effects model was used in all analyses due to the expected heterogeneity among the studies and the slightly varied measurement methodologies they used. Univariate meta‐regression was also employed to explore the sources of heterogeneity among the studies and identify potential variables for determining BDNF levels. Bubble plots were created to visualize the correlation between these variables and the SMDs.

The mean and standard deviation were estimated using methods devised by Luo, Wan, and Shi when the median and interquartile range, or mean and range, were provided (Wan et al. 2014; Luo et al. 2018; Shi et al. 2023, 2020). The *I^2^* and tau^2^ statistics were used to assess heterogeneity in all meta‐analyses. A result was deemed statistically significant if its *p* value was below 0.05 and its *I* ^2^ value exceeded 50%.

## 3 Results

### 3.1 Study Characteristics

**FIGURE 1:**
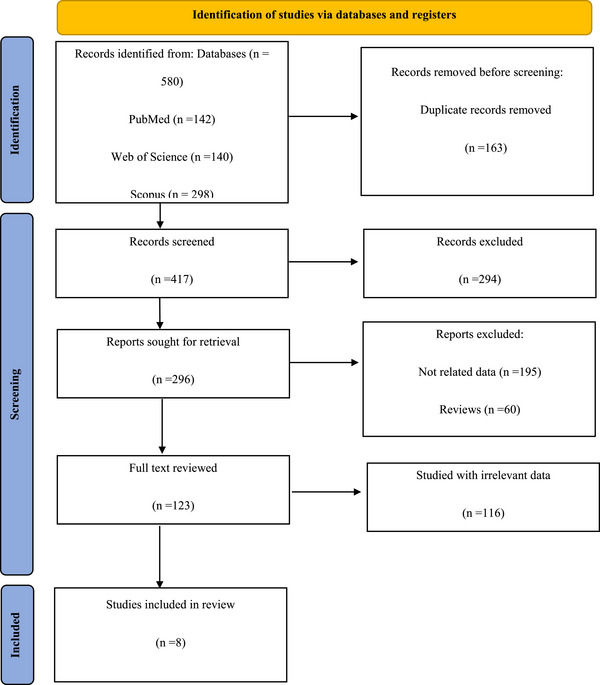
Flow PRISMA diagram representing the selection process of eligible studies.

Our search yielded a total of 508 records retrieved from PubMed (*n* = 142), Web of Science (*n* = 140), and SCOPUS (*n* = 298). After eliminating duplicate studies and excluding reports not meeting our study criteria, we identified 8 studies for inclusion in this review, encompassing a total of 313 glioma patients and 194 controls. A PRISMA flow chart outlining the study selection process is depicted in Figure 1. Detailed information regarding the included studies is presented in Table 1. All studies were assessed as “good” or “very good” based on their NOS quality assessment scores, as outlined in Table S2.

**TABLE 1:** Characteristics of studies evaluating the relation between BDNF levels in glioma.

Author	Year	Design	Location	Glioma	Control	N. Control	N. Glioma	Source	% female glioma	% female control	BDNF concentration Glioma	BDNF concentration control	*p* value	Main findings
Wójtowicz et al. (2023)	2023	Case‐control	Poland	Glioblastomas	elective brain surgery for an unrelated, nonneoplastic, non‐traumatic pathology	19	24	CSF Plasma Tumor Tissue	7	16	6.5 pg/mL 288.59 pg/mL 14.23 ng/g	11.48 pg/mL 574.06 —	0.002 0.0005 —	CSF and plasma BDNF levels were significantly lower in glioma patients than in the control group. The level of BDNF in tumor tissue did not differ significantly depending on IDH1 mutation status or other factors such as age, smoking, or medication use.
Kluckova et al. (2023)	2023	Case‐control	Slovakia	Grade IV glioma	healthy volunteers without any personal or family history of cancer or acute inflammatory disease, and they were randomly recruited from a larger population sample	17	30	Plasma	50	50	621 pg/mL (IQR: 473.2)	1265 pg/mL (IQR 681.77)	0.001	Plasma levels of BDNF were significantly lower in glioblastoma patients compared to healthy controls. BDNF, along with sTREM‐1 and sHLA‐G, demonstrated a good discriminatory capability for glioblastoma patients, with the best performance achieved when combined (AUC = 0.9534, sensitivity 79.2%, specificity 94.1%). In grade IV glioma, BDNF levels were significantly decreased, suggesting its potential as a diagnostic marker.
				Grade III glioma		17	11	36	50	863.6 pg/mL (SD: 510)	1270.3 pg/mL (SD: 525)	0.05	
				Grade II glioma		17	19	26	50	1021.3 pg/mL	1270.3 pg/mL	0.18	
Zheng and Chen (2020)	2020	Case‐control	China	_	normal brain tissue	23	23	Tissue	52				0.0001	BDNF is significantly upregulated in glioma tissues and cells, correlating with higher clinical grading in glioma patients. Silencing BDNF inhibits glioma cell proliferation, migration, and invasion while promoting apoptosis. Furthermore, miR‐489‐3p negatively regulates BDNF expression through direct interaction, with reduced miR‐489‐3p levels observed in glioma tissues and cells, indicating its potential as a therapeutic target for glioma.
Xiong et al. (2015)	2015	Case‐control	China	Grades I and II glioma Grades III and IV glioma	nonneoplastic brain tissues	10 10	21 21	Tissue	62 38	20				Mature BDNF and TrkB expression levels escalate with glioma malignancy grade, confirmed by immunostaining, RT‐qPCR, and ELISA (p < 0.001). Their positive correlation with malignancy grade (*p* < 0.001) suggests their involvement in glioma progression.
Lange et al. (2014)	2014	Case‐control	USA	Grade IV glioma Grade II glioma	Healthy adults without a history of brain tumors and without neurological symptoms who were presumed not to have a glioma.	15 15	23 11	Plasma	43 64					Mature BDNF expression is significantly increased in high‐grade glioma tissues compared to low‐grade gliomas and control tissues. Similarly, TrkB expression is markedly elevated in high‐grade gliomas. A positive association was found between mature BDNF and TrkB expression levels with the malignancy grade of glioma, indicating a potential role in glioma progression.
Xiong et al. (2013)	2013	Case‐control	China	Low‐grade glioma High‐grade glioma	Nonneoplastic brain tissues from patients subjected to lobe resection for epilepsy surgery, brain trauma, hypertensive cerebral hemorrhage, and internal decompression	13 13	19 12	Tissue			1.21±0.16 (mean, SE) 1.81+0.15 (mean, SE)	0.92 ± 0.15 (mean, SE)		ProBDNF expression intensifies with glioma malignancy, predominantly in high‐grade tumors. Moreover, p75NTR expression parallels proBDNF, showing increased levels in high‐grade gliomas. Correlation analysis indicates positive associations between proBDNF, p75NTR, and malignancy grade. The proBDNF to mature BDNF ratio decreases with increasing tumor grade, suggesting a potential role in glioma progression. ProBDNF treatment induces morphological changes and differentiation in C6 glioma cells, potentially mediated through p75NTR activation.
Chiaretti et al. (2004)	2004	Case‐control	Italy	Low‐grade glioma	Children of similar age, gender, and weight who had surgical interventions for either cerebral vascular condition	9	10	Tissue CSF	30	—				BDNF levels were found to increase in the CSF of affected children, suggesting distinct correlations between neurotrophic factors and the biology of low‐grade astrocytomas and ependymomas in childhood brain neoplasms.
Ilhan‐Mutlu et al. (2013)	2013	Case‐control	Austria	Grade II glioma Grade III glioma Grade IV glioma	Twenty‐six healthy volunteers and 25 patients with multiple sclerosis (MS) as nonmalignant central nervous system pathology served as control subjects.	61 61 61	7 10 34	Plasma	43 60 41	HC:42, MS:72 HC:42, MS:72 HC:42, MS:72	30.6 (0–76) ng/mL (median, min‐max) 0 (0–74.2) ng/mL 8.2 (0–82) ng/mL	Not performed Not performed Not performed		Plasma concentrations of BDNF were significantly lower in ICM patients compared to glioma patients. However, BDNF did not show a statistically significant association with neuroradiological parameters, tumor location, or other investigated markers.

### 3.2 Plasma Levels of BDNF Compared to Controls

**FIGURE 2:**
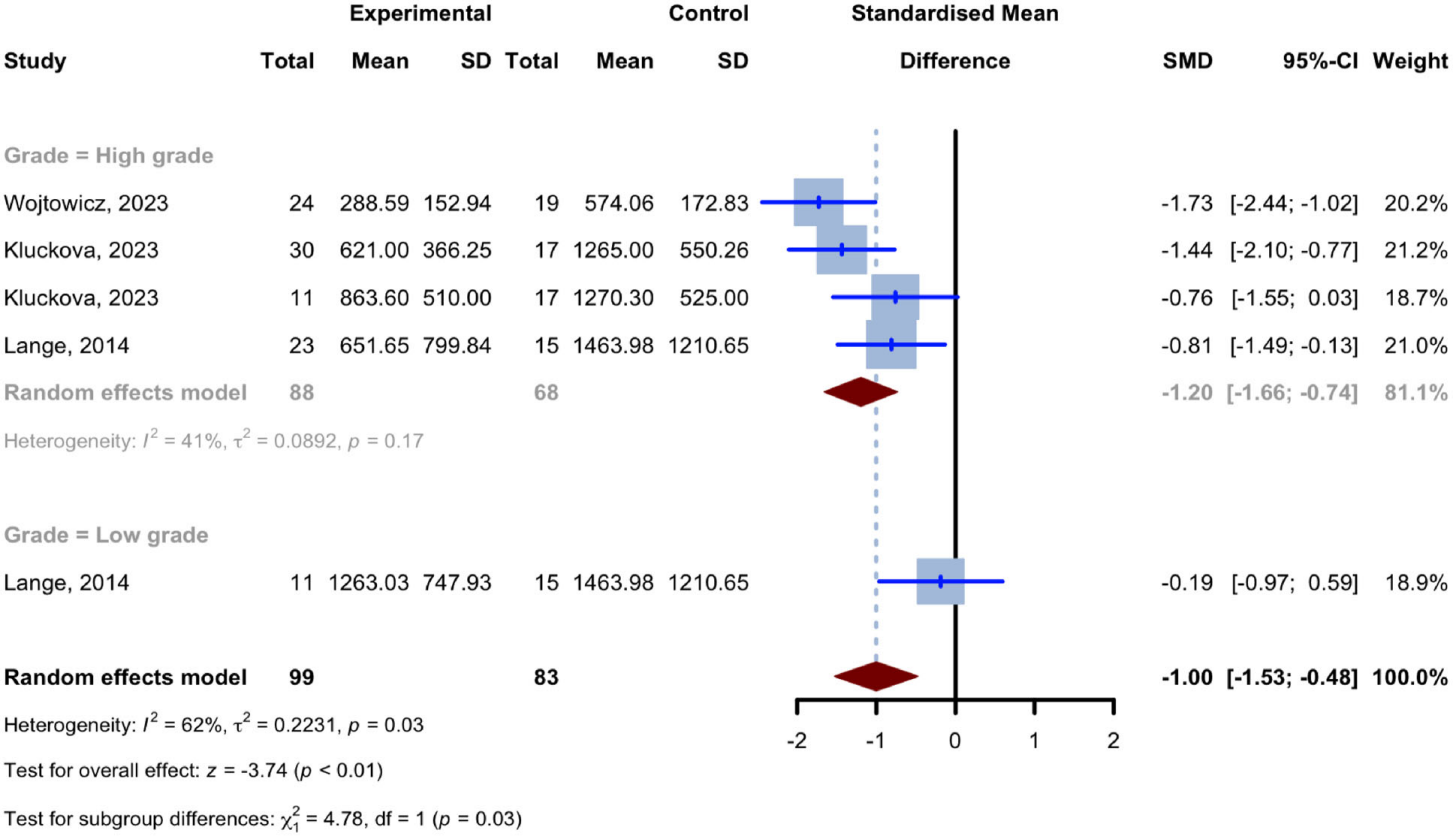
Meta‐analysis of glioma patients’ plasma levels of BDNF in comparison to controls.

Three studies covering five comparisons between groups of glioma patients and healthy controls examined the plasma levels of BDNF. The comparisons included a total of 99 cases with glioma and 51 healthy controls. Although Ilhan‐Mutlu et al. (2013) conducted a study on BDNF plasma levels, we did not include their data in their meta‐analysis since they did not report the BDNF levels of their control group. Across all comparisons, glioma cases had a reduced mean plasma BDNF concentration compared to the control group. The combined analysis of the five plasma BDNF comparisons yielded a pooled SMD of −1.0026 (95% CI: [−1.5284, −0.4769]; *p* = 0.0002; *I* ^2^ = 61.8%; Figure 2).

When considering individuals with high‐grade glioma (Grade III or IV) and healthy controls, there were four investigations from three studies: Wójtowicz et al. (2023), Kluckova et al. (2023), and Lange et al. (2014). The comparisons involved 88 cases of glioma and 51 healthy controls. The combined SMD for the high‐grade glioma subgroup was −1.1964 (95% CI: [−1.6564, −0.7364]; *I* ^2^ = 40.8%). The subgroup for comparing individuals with low‐grade gliomas (Grade I or II) to healthy controls consisted of a single comparison from Lange et al. (2014), which compared 11 low‐grade glioma (Grade I or II) patients and 15 healthy controls with an SMD of −0.1866 (95% CI: [−0.9665, 0.5934]). There was a meaningful difference between the SMDs of high‐grade and low‐grade glioma subgroups, as demonstrated by the statistically significant results of the test for subgroup differences (high‐grade subgroup vs. low‐grade subgroup; *p* = 0.0288).

**FIGURE 3:**
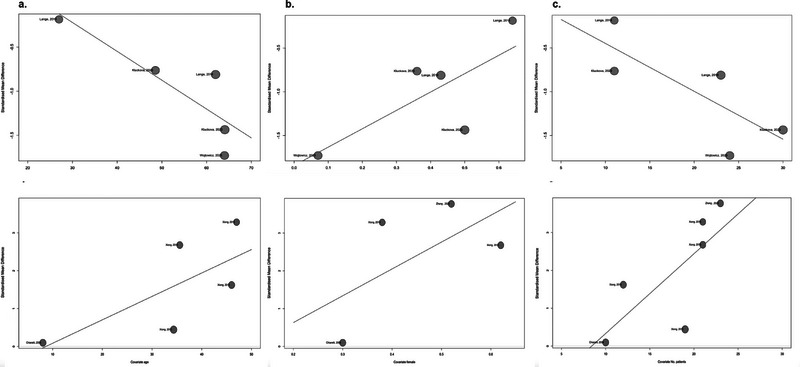
Univariate meta‐regression bubble plots: (a) patients age as a covariate in plasma, (b) female percentage as a covariate in plasma, (c) number of patients as a covariate in plasma, (d) patients’ age as a covariate in tissue, (e) female percentage as a covariate in tissue, and (f) number of patients as a covariate in tissue.

The univariate meta‐regression analysis, using the SMDs as the outcome variable and the age of patients as a covariate, showed a statistically significant correlation (estimate of −0.0327, *p* = 0.0085, *R* ^2^ = 95.41%). These findings suggest that there is an inverse correlation between the age of patients and the SMD reported in the studies. The number of glioma patients in each study as a covariate was also found to have a statistically significant association with the reported SMDs (estimate of −0.0558, *p* = 0.0311, *R* ^2^ = 76.75%). These studies suggest a negative correlation between the number of patients and the reported SMD. The percentage of females as a covariate did not show a statistically significant association with the SMDs (estimate of 2.1082, *p* = 0.0637, *R* ^2^ = 58.72%) (Table 2, Figure 3).

**TABLE 2:** Univariate meta‐regression.

Plasma samples SMD meta‐regression						
Covariate	No. of studies	No. of patients	Meta‐regression estimate	95% CI	*R* ^2^	*p* value
Patients age	5	99	−0.0327	−0.0570 to −0.0083	95.41%	0.0085
Female %	5	99	2.1082	−0.1204 to 4.3368	58.72%	0.0637
No. of patients	5	99	−0.0558	−0.1065 to −0.0051	76.75%	0.0311
**Tissue samples SMD meta‐regression**						
**Covariate**	**No. of studies**	**No. of patients**	**Meta‐regression estimate**	**95% CI**	***R* ^2^**	***p* value**
Patients age	5	83	0.0619	−0.0075 to 0.1312	38.15%	0.0803
Female %	4	75	7.1035	−5.4604 to 19.6674	9.11%	0.2678
No. of patients	5	106	0.2107	0.0205 to 0.4009	46.12%	0.0299
**Glioma patients mean plasma BDNF**						
Covariate	**No. of studies**	**No. of patients**	**Meta‐regression estimate**	**95% CI**	***R* ^2^**	***p* value**
Age	5	99	−0.0281	−0.0527 to −0.0036	54.11%	0.0244
Female %	5	99	2.2897	0.9722 to 3.6073	77.90%	0.0007

#### 3.2.1 Mean Plasma Levels of BDNF

**FIGURE 4:**
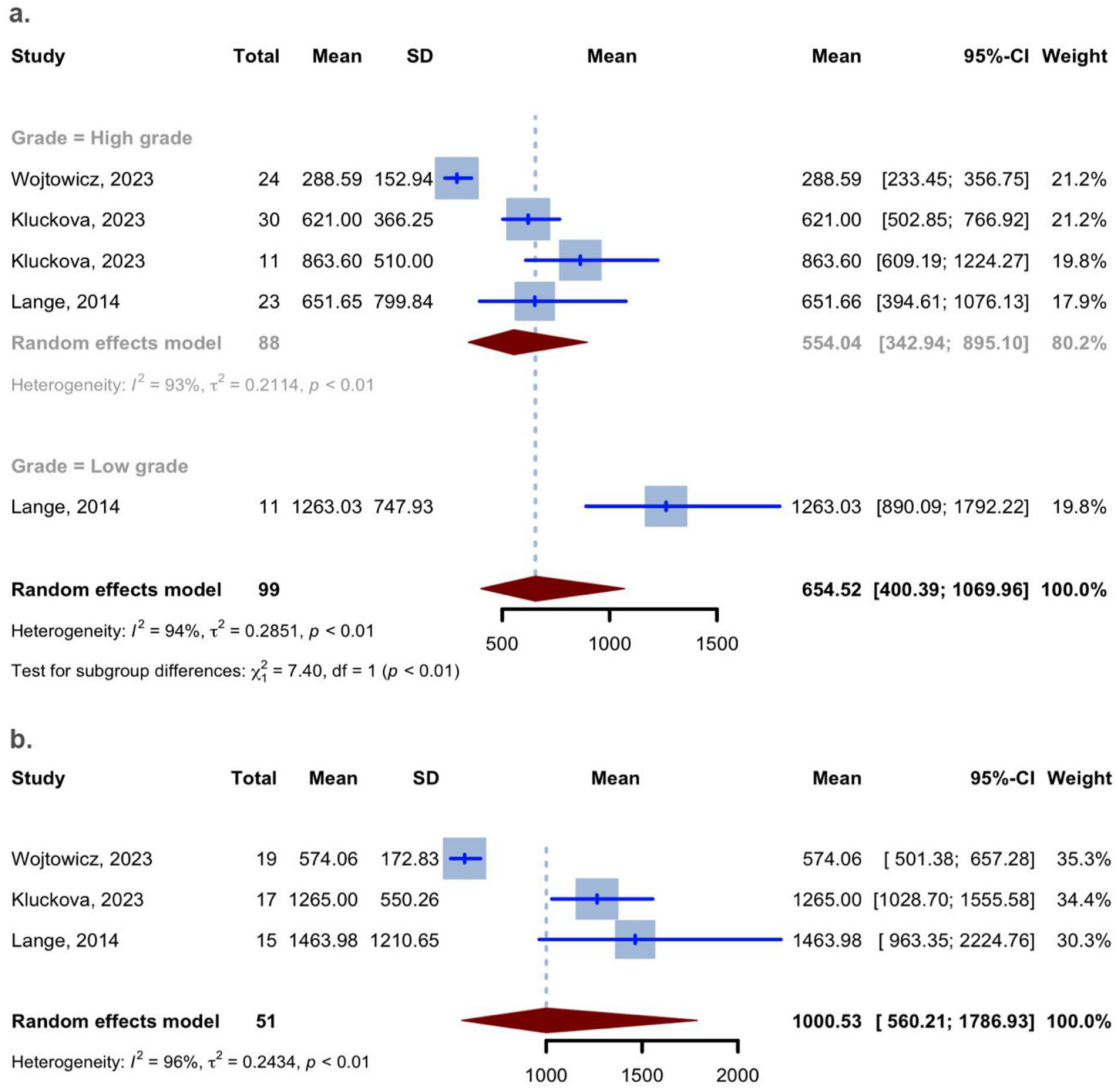
Meta‐analysis of mean plasma levels of BDNF: (a) plasma levels of BDNF in patients with gliomas and (b) plasma levels of BDNF in healthy controls.

The studies on plasma levels of BDNF presented their findings in a way that allowed for a meta‐analysis of the mean, as the units used were compatible. The average plasma level of BDNF in all 99 glioma patients, regardless of the grade, was 654.52 pg/mL (95% CI: [400.39, 1069.96]; *I* ^2^ = 93.9%; Figure 4). The average plasma level of BDNF in the 51 healthy controls was 1000.53 pg/mL (95% CI: [560.21, 1786.93]; *I* ^2^ = 95.9%). The average plasma level of BDNF in all 88 high‐grade glioma patients (Grade IV or III) was 554.04 pg/mL (95% CI: [342.94, 895.10]; *I* ^2^ = 92.5%; Table 3).

**TABLE 3:** Mean plasma levels of BDNF.

Plasma samples						
	No. studies	No. individuals	Mean (pg/mL)	95%CI	*I* ^2^	Mean difference
Control group	3	51	1000.5256	560.2059; 1786.9348	95.9%	Ref.
High grade (IV or III)	4	88	554.0412	342.9375; 895.0953	92.5%	−446.4844
Total	5	99	654.5238	400.3915; 1069.9564	93.9%	−346.0018

**FIGURE 5:**
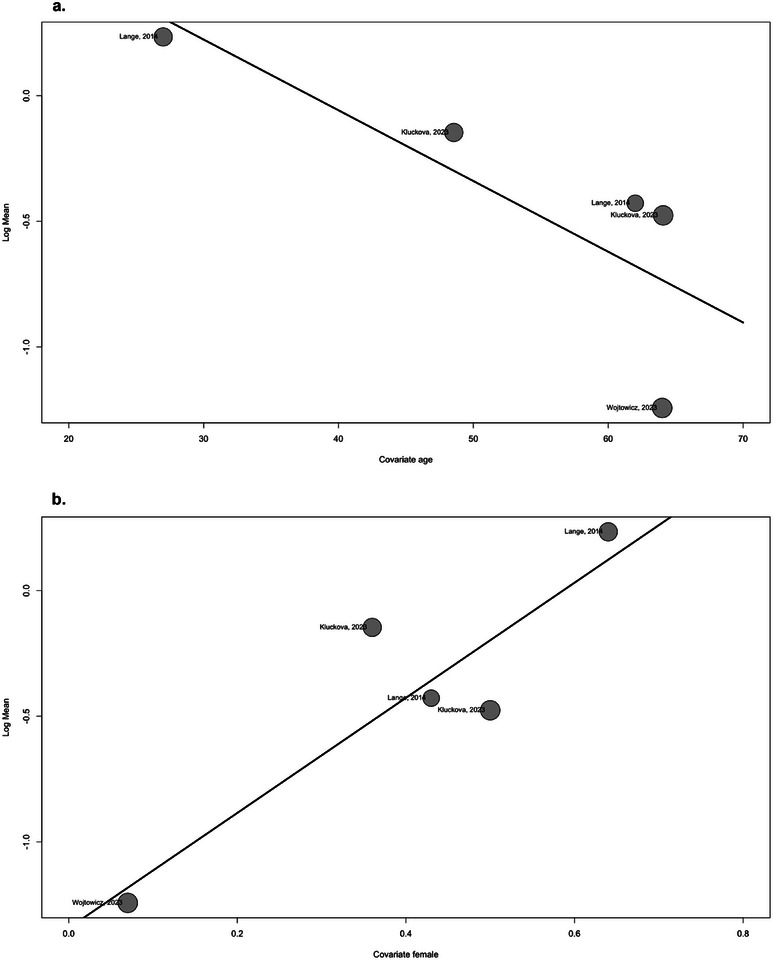
Univariate meta‐regression bubble plots: (a) age as a covariate and (b) female percentage as a covariate.

The univariate meta‐regression analysis revealed a significant association in glioma patients between the plasma mean levels of BDNF (outcome variable) and both age and female (covariates). The analysis showed a negative correlation for patients aged 0 (estimate of −0.0281, *p* = 0.0244, *R* ^2^ = 54.11%) and a positive correlation for female percentage (estimate of 2.2897, *p* = 0.0007, *R* ^2^ = 77.90%; Table 2, Figure 5).

### 3.3 Tissue Levels of BDNF Compared to Controls

**FIGURE 6:**
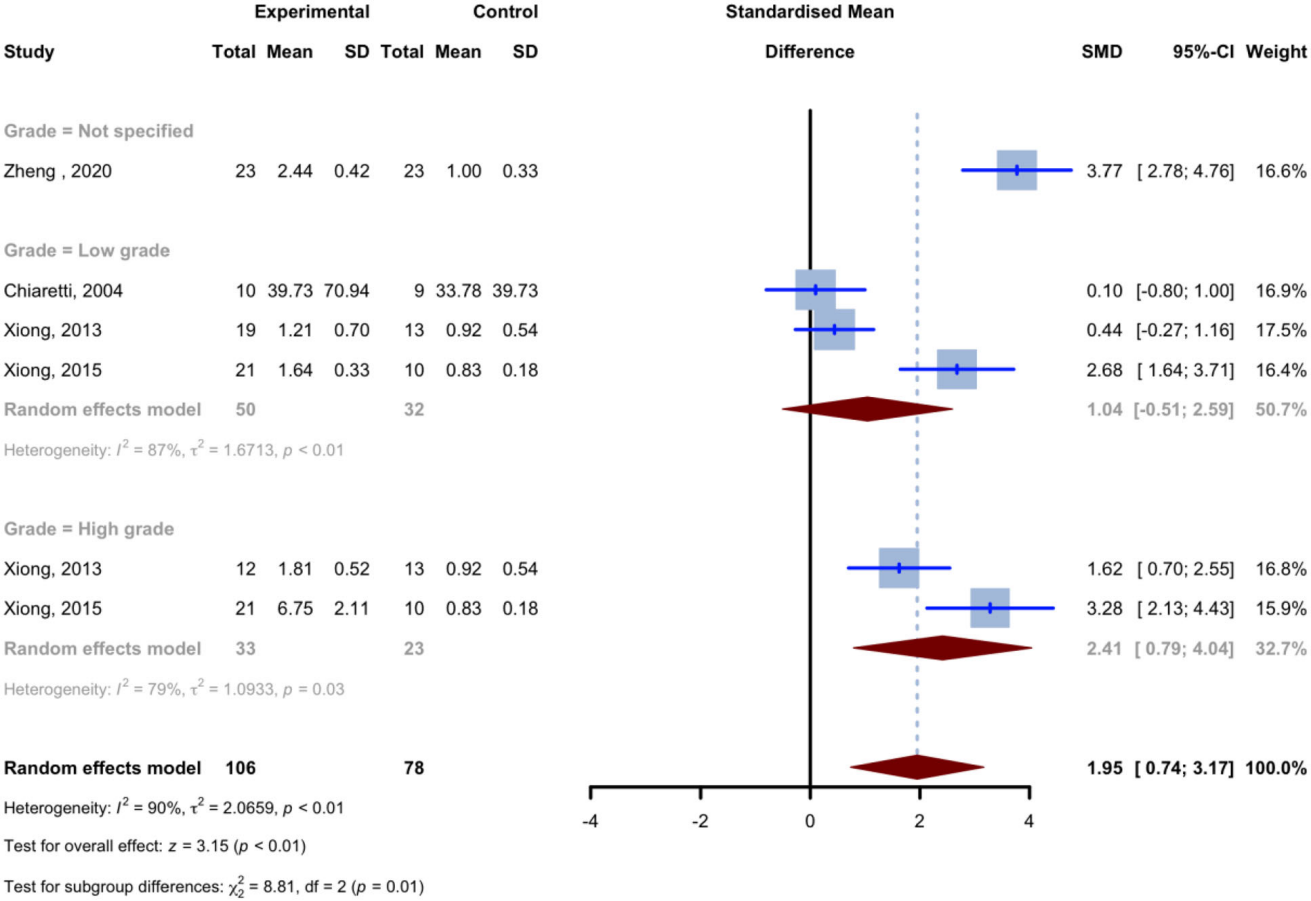
Meta‐analysis of tissue levels of BDNF in patients with glioma compared to controls.

Six comparisons from four studies reported differences in tissue levels of BDNF in glioma patients compared to healthy controls. The studies included 106 glioma cases and 55 healthy controls. In all comparisons, glioma cases had higher average tissue levels of BDNF compared to the control group. For all six tissue BDNF comparisons combined, the pooled SMD was 1.9513 (95% CI: [0.7365, 3.1661], *p* = 0.0016; *I* ^2^ = 90.3%; Figure 6). The comparison of individuals with high‐grade glioma (Grade III or IV) with healthy controls included two investigations from the investigations conducted by Xiong, Zhou, Yang, et al. (2013) and Xiong et al. (2015). It encompassed 33 cases of glioma and 23 healthy controls. The combined SMD for the high‐grade glioma subgroup was 2.4140 (95% CI: [0.7894, 4.0386]; *I* ^2^ = 79.4%). The comparison of individuals with low‐grade gliomas (Grade I or II) to healthy controls consisted of three comparisons conducted by Chiaretti et al. (2004), Xiong, Zhou, Yang, et al. (2013), and Xiong et al. (2015). It included 50 cases of glioma and 32 controls. The combined SMD for the low‐grade glioma subgroup was 1.0444 (95% CI: [−0.5060, 2.5948]; *I* ^2^ = 87.4%). Although the Zheng and Chen (2020) study stated that they were investigating glioblastoma patients (which is glioma Grade IV by definition) for BDNF tissue levels, they also stated that the patients varied in Grades from I to IV. To avoid bias, we assigned this comparison to a third separate subgroup (not specified grade gliomas). The SMD for not‐specified‐grade glioma subgroups was 3.7707 (95% CI: [2.7806, 4.7608]). The test for comparing subgroups of high‐grade gliomas, low‐grade gliomas, and not‐specified‐grade gliomas found statistically significant differences in SMDs of these subgroups (*p* = 0.0122).

The univariate meta‐regression analysis, utilizing the SMDs as the outcome variable and incorporating the age of patients (*p* = 0.0803) and percentage of females (*p* = 0.2678) as covariates, did not reveal a statistically significant correlation. The analysis showed a positive correlation for the number of glioma patients (estimate of 0.2107, *p* = 0.0299, *R* ^2^ = 46.12%, Figure 3).

### 3.4 CSF Levels of BDNF Compared to Controls

The reported findings regarding BDNF levels in the CSF of glioma patients compared to controls are somewhat contradictory. Wójtowicz et al. (2023) and Chiaretti et al. (2004) both conducted a comparative analysis of BDNF levels in the CSF of glioma patients compared to healthy individuals. Wójtowicz et al. conducted a study that analyzed 24 CFS samples from patients with gliomas and 19 CFS samples from healthy controls. The study focused on patients diagnosed with glioblastoma, a type of high‐grade glioma. Wójtowicz et al. found that patients with glioblastoma had a lower mean CSF BDNF level of 6.50 pg/mL compared to the control group of 11.48 pg/mL. We calculated an SMD of −0.91 (95% CI: [−1.54, −0.27]) for this study, which also suggests a reduced BDNF level in CFS samples of glioma patients. Chiaretti et al. conducted a study that analyzed 10 CFS samples from glioma patients and 9 CFS samples from healthy controls. The study specifically focused on individuals diagnosed with low‐grade gliomas. Chiaretti et al. reported that individuals diagnosed with low‐grade glioma exhibited a significantly elevated CSF BDNF level of approximately 605 pg/mL, in contrast to the control group, which had an average level of around 200 pg/mL. We calculated an SMD of 1.29 (95% CI: [0.28, 2.30]) for this study, which also suggests an elevated BDNF level in CFS samples of glioma patients.

### 3.5 Publication Bias

**FIGURE 7:**
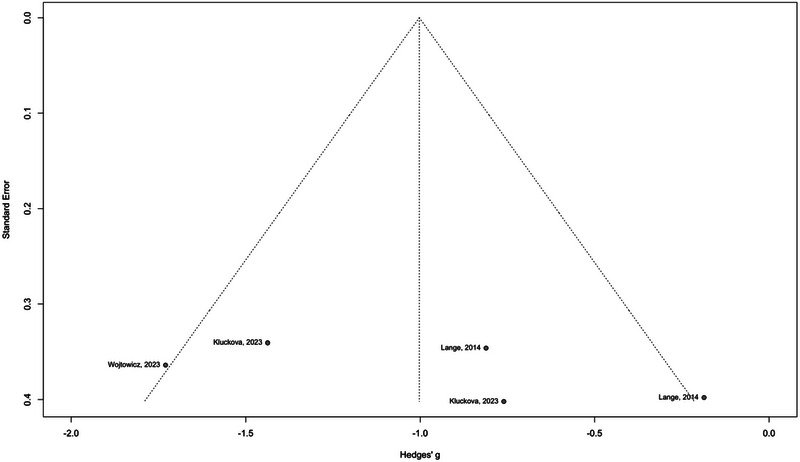
Funnel plot asymmetry analysis for plasma samples.

**FIGURE 8:**
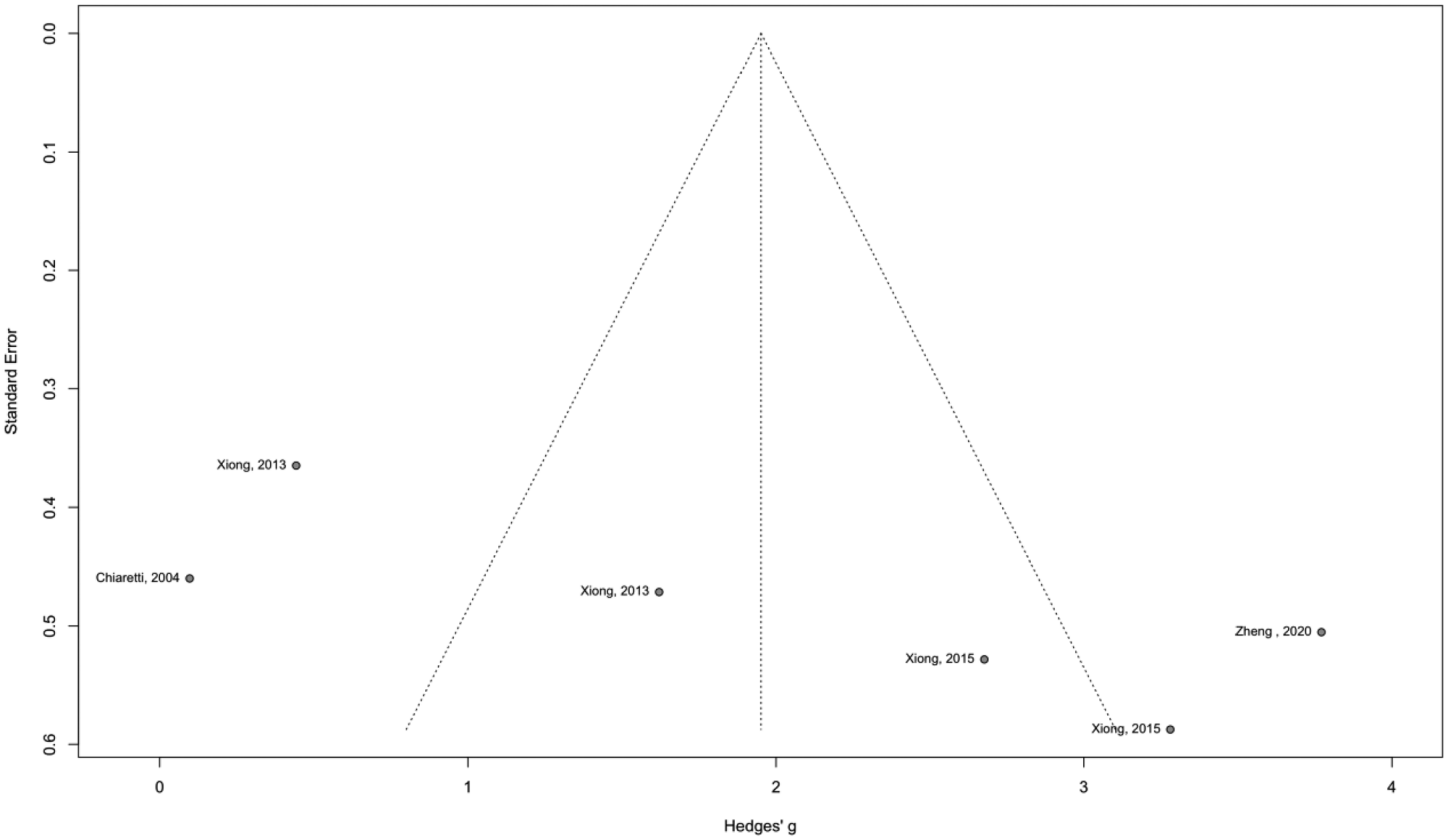
Funnel plot asymmetry analysis for tissue samples.

The publication bias assessment was conducted for both plasma and tissue samples. For plasma samples, the rank correlation test of funnel plot asymmetry yielded a *p* value of 0.3272, suggesting no significant evidence of publication bias. Similarly, the Egger linear regression test also indicated no significant funnel plot asymmetry with a *p* value of 0.2907 (Figure 7). Conversely, for tissue samples, while the Egger linear regression test demonstrated an insignificant result with a *p* value of 0.0675, the rank correlation test yielded a *p* value of 0.0388, indicating significant asymmetry and suggesting potential publication bias in studies examining tissue samples (Figure 8).

## 4 Discussion

The meta‐analysis showed that glioma cases have significantly reduced BDNF plasma concentrations compared to controls, with lower levels in higher pathological grades. In contrast, patients suffering from glioma have higher tissue concentrations compared to healthy controls, with higher levels in higher pathological grades.

It is well established that TrkB, a key regulator of malignancies affecting neuroplasticity, is considerably elevated in more invasive tumors (Serafim Junior et al. 2020; Taylor et al. 2023). BDNF expression has also been associated with various TrkB‐expressing cancers, including those of the nervous system. Previous studies have shown that BDNF mRNA is a common transcript in cell lines derived from gliomas (Hamel et al. 1993). BDNF is not a glioma‐specific protein as it is physiologically expressed in the cerebral cortex, hippocampus, basal forebrain, retina, kidneys, and saliva (Frota et al. 2009; Di Lazzaro et al. 2007; Saruta et al. 2012; Morita, Lee, and Her 2009). The precursor is proteolytically converted to the mature form of BDNF and a propeptide (proBDNF) in the CNS, which function oppositely (Sun et al. 2012; Koshimizu et al. 2010; Teng et al. 2010). The mature BDNF is involved in angiogenesis and tumorigenesis by targeting TrkB, modulating the proliferation, survival, and differentiation of neural stem cells (Ji et al. 2014, 2015). Thus, inhibiting BDNF could impair tumor cell survival and increase sensitivity to chemotherapy (Ho et al. 2002). TrkB mRNA expression was also shown to be significantly lower in patients with low‐grade gliomas compared to high‐grade (Xiong et al. 2015). In contrast, a prior study has exhibited the dose‐dependent efficacy of exogenous proBDNF in triggering apoptosis (Yang et al. 2017). ProBDNF, a potent tumor suppressor, binds to the p75 neurotrophin receptor (NTR) with high affinity, reducing the migration and growth of glioma cells (Lu, Pang, and Woo 2005). Thus, attentively modulating the balance between mature BDNF and proBDNF levels could promisingly affect glioma progression as a potential therapeutic target.

Our meta‐analysis showed that glioma cases have significantly reduced BDNF plasma concentrations compared to controls, with lower levels in higher pathological grades, suggesting for the first time that plasma BDNF could be both a diagnostic and a prognostic marker in glioma. In contrast to our findings, Chiaretti et al. (2004), comparing the concentration of all neurotrophins among children with low‐grade gliomas, found no statistically significant difference in plasma levels of BDNF. However, consistent with our results, other studies have illustrated that plasma concentrations of BDNF were lower in glioblastoma patients (Wójtowicz et al. 2023; Kluckova et al. 2023). Lange et al. (2014) found that glioma patients have lower plasma levels of BDNF than controls, but the authors concluded that BDNF would not be a promising plasma marker. Although Lang et al. reported lower levels of BDNF in glioma patients with grades II and IV compared to controls, they did not compare BDNF levels among low‐ and high‐grade patients. However, our findings showed significant plasma concentration differences between high‐ and low‐grade gliomas, suggesting BDNF as a potential marker for glioma grading.

The variations in BDNF levels observed in our study may be due to differences in the populations studied, variations in the ages of the patients, differences in the gender distribution of the populations, and, most importantly, the source of BDNF. A randomized controlled study conducted on a population with obesity revealed that the levels of circulating BDNF were higher in women at baseline. Furthermore, the study found a strong correlation between BDNF levels, diet, and exercise (Glud et al. 2019). Studies have also suggested that the menstrual cycle may influence BDNF plasma levels along with hormonal alterations (Cubeddu et al. 2011). In contrast, Hayley et al. (2015) reported no difference in hippocampal BDNF between the sexes of humans. Lommatzsch et al. (2005) also reported no considerable difference between genders in BDNF levels. Our findings demonstrated a significant difference in BDNF levels between sexes in glioma patients, as well as a significant inverse correlation between patient age and the pooled plasma concentration of BDNF. Confirming our findings, age has been shown to have an influence on circulating BDNF concentrations in peripheral blood by other studies (Lommatzsch et al. 2005).

Plasma BDNF levels have been observed to decrease with advancing age or increased body weight. It is also shown that platelet BDNF, which is obtained from external sources during platelet formation, is not affected by age or weight (Fujimura et al. 2002). Platelets also acquire BDNF from organs such as neurons or endometrium (Karege, Schwald, and Cisse 2002; Lommatzsch et al. 1999). Thus, platelet‐derived BDNF could show organ production of BDNF is influenced by gender. There is still a lack of systematic and comprehensive comparison of the sex‐specific alteration of BDNF concentrations among glioma patients. It is important to mention that the included studies in the current review did not measure the levels of platelet BDNF. Based on imbalances caused by age, weight, population, and gender in plasma BDNF, as well as the low levels of BDNF observed in peripheral blood, we propose that future research should concentrate on examining platelet BDNF levels in addition to plasma levels to elucidate the impact of neural production. Further investigations should also focus on cut‐off points and the specificity and sensitivity of this biomarker in different grades of glioma.

Klein et al. (2011) suggested that plasma BDNF levels were correlated with brain‐tissue BDNF levels. It has been known that TrkB has been significantly upregulated in more invasive tumors (Assimakopoulou et al. 2007). An in vitro study has demonstrated that when patient‐derived cultures are exposed to BDNF, it results in increased proliferation of tumor cells (Venkatesh et al. 2015). Various in vitro studies have also reported that tissue BDNF levels were increased in glioblastoma patients, hypothesizing tissue BDNF as a potential diagnostic biomarker (Jones et al. 2021). BDNF‐associated protein and mRNA have also been shown to be increased in glioma tissues compared to healthy brain tissues (Xiong et al. 2015; Xiong, Zhou, Lim, et al. 2013; Wang, Liu, and Song 2018). Consistent with previous findings, we also found higher tissue concentrations of BDNF in glioma tissues compared to the control group. Our findings also indicated a difference in BDNF levels between high‐ and low‐grade glioma tissues. Yan, Yu, and Li (2009) previously reported a gradual upregulation of BDNF in human glioma tissue in association with higher WHO grades. Our findings also indicated that there is no correlation between gender and age and the differences in tissue BDNF levels between patients and controls. Overall, our findings underline the potential of tissue BDNF as a biomarker for glioma diagnosis and prognosis; however, cut‐off values, specificity, and sensitivity remain vague.

To the best of our knowledge, this is the first study evaluating concentrations of BDNF from different sources in patients suffering from glioma. However, the current study has a number of limitations. First, the limited number of studies with small sample sizes investigating the significance of this neurotrophic factor in glioma presented substantial obstacles to drawing additional conclusions. Second, alterations in BDNF levels can be impacted by numerous confounding variables, such as psychological disorders, tumor grade, age, gender, analgesic usage, and other bodily secretions, which could not be further addressed in this study. The effect of these confounding factors on BDNF levels was not sufficiently examined in the studies that were analyzed, highlighting the necessity for further investigation. Furthermore, the studies that were included did not evaluate the levels of mature BDNF/proBDNF or platelet BDNF. Moreover, it is necessary to examine the correlation between BDNF derived from plasma and tissues, indicating the requirement for further investigation.

## 5 Conclusion

The present study underscored the potential of BDNF as a diagnostic and prognostic biomarker in glioma. We observed significant differences in both plasma and tissue levels of BDNF between glioma patients and healthy controls, with glioma patients having lower plasma concentrations and higher tissue concentrations. Notably, our findings suggested an inverse correlation between patient age and plasma BDNF levels, highlighting age as a potential influencing factor. Female gender also correlated positively with plasma BDNF levels. Our study also emphasized the clinical relevance of BDNF in glioma grading and highlighted its potential for improving diagnostic accuracy and prognosis assessment.

## Author Contributions

**Fatemeh Hasani**: conceptualization, investigation, methodology, validation, writing–original draft, writing–review and editing, project administration, data curation, resources, supervision. **Mahdi Masrour**: conceptualization, investigation, writing–original draft, software, data curation, resources, methodology. **Sina Khamaki**: conceptualization, investigation, writing–original draft, data curation, software. **Kimia Jazi**: conceptualization, validation, writing–original draft. **Erfan Ghoodjani**: investigation, writing–original draft, methodology. **Antonio L. Teixeira**: writing–review and editing, data curation, project administration.

## Ethics Statement

The authors have nothing to report.

## Conflicts of Interest

The authors declare no conflicts of interest.

### Peer Review

The peer review history for this article is available at https://publons.com/publon/10.1002/brb3.70266.

## Supporting information



Supporting Information

## Data Availability

All data used for this manuscripts is included in the main or supplementary files.
