# Supplementary material for: Brain‐Derived Neurotrophic Factor (BDNF) as a Potential Biomarker in Brain Glioma: A Systematic Review and Meta‐Analysis
Source: Brain Behav. 2025 Jan 9;15(1):e70266. doi: 10.1002/brb3.70266 (PMC11726635; doi:10.1002/brb3.70266)
Supplement: Supplementary file 1 — Supporting Information [file BRB3-15-e70266-s001.docx]

**Supplementary materials**

**Supplementary Table 1.** The search queries used for each database and the search results

|  | **Query** | **Results (No.)**  **December 22, 2023** |
| --- | --- | --- |
| **PubMed** | | |
| #1 | ("Brain-Derived Neurotrophic Factor"[Mesh] OR "BDNF"[tiab] OR "brain-derived neurotrophic factor"[tiab] OR "brain-derived neurotrophic factor"[tiab]) | 31,149 |
| #2 | (((((glioma*[Title/Abstract]) OR (glial cell tumor*[Title/Abstract])) OR (glioblastoma*[Title/Abstract])) OR (malignant glioma[Title/Abstract])) | 105,725 |
| #3 | #1 AND #2 | 142 |
| **Web of Science** | | |
| #1 | TS=(“Brain-Derived Neurotrophic Factor*” OR “BDNF*” OR “brain-derived neurotrophic factor*” OR “brain-derived neurotrophic factor*”) | 28,771 |
| #2 | TS=(“glioma*” OR “glial cell tumor*” OR “glioblastoma*” OR “malignant glioma”) | 93,597 |
| #3 | #1 AND #2 | 140 |
| **SCOPUS** | | |
| #1 | (TITLE-ABS-KEY("Brain-Derived Neurotrophic Factor") OR TITLE-ABS-KEY("BDNF") OR TITLE-ABS-KEY("brain-derived neurotrophic factor") OR TITLE-ABS-KEY("brain-derived neurotrophic factor")) | 49,992 |
| #2 | (TITLE-ABS-KEY(“glioma*” OR “glial cell tumor*” OR “glioblastoma*” OR “malignant glioma”)) | 157,580 |
| #3 | #1 AND #2 | 298 |
| **Total** | | 580 |
| **Total without duplicates** | | 417 |

**Supplementary Table 2.** Quality scores of included studies using Newcastle-Ottawa Scale (maximum score of 9)

| Author, year | Selection | | | | Comparability | Exposure | | | Overall score |
| --- | --- | --- | --- | --- | --- | --- | --- | --- | --- |
|  | Case definition | Representativeness | Selection of Controls | Definition of Controls |  | Ascertainment of exposure | Same method of ascertainment | Non-Response rate |  |
| Wójtowicz, 2023 (1) | * | * |  | * | ** | * | * | * | 8 |
| Kluckova, 2023 (2) | * | * | * | * | ** | * | * | * | 9 |
| Zheng, 2020 (3) | * | * | * | * | ** | * | * | * | 9 |
| Xiong, 2015 (4) | * | * |  | * | ** | * | * | * | 8 |
| Lange, 2014 (5) | * | * |  | * | ** | * | * | * | 8 |
| Xiong, 2013 (6) | * | * |  | * | ** | * | * | * | 8 |
| Ilhan-Mutlu, 2013 (7) | * | * | * | * | ** | * | * | * | 9 |
| Chiaretti (8) | * | * |  | * | ** | * | * | * | 8 |

1. Wójtowicz K, Czarzasta K, Przepiorka L, Kujawski S, Cudnoch-Jedrzejewska A, Marchel A, et al. Brain-Derived Neurotrophic Factor (BDNF) Concentration Levels in Cerebrospinal Fluid and Plasma in Patients With Glioblastoma: A Prospective, Observational, Controlled Study. Cureus. 2023;15(11):e48237.

2. Kluckova K, Kozak J, Svajdler M, Steno J, Matejcik V, Durmanova V, et al. BDNF, sHLA-G, and sTREM-1 are useful blood biomarkers for identifying grade IV glioma patients. Neoplasma. 2023;70(1):166-76.

3. Zheng B, Chen T. MiR-489-3p inhibits cell proliferation, migration, and invasion, and induces apoptosis, by targeting the BDNF-mediated PI3K/AKT pathway in glioblastoma. Open Life Sciences. 2020;15(1):274-83.

4. Xiong J, Zhou LI, Lim Y, Yang M, Zhu YH, Li ZW, et al. Mature brain-derived neurotrophic factor and its receptor TrkB are upregulated in human glioma tissues. Oncol Lett. 2015;10(1):223-7.

5. Lange RP, Everett A, Dulloor P, Korley FK, Bettegowda C, Blair C, et al. Evaluation of eight plasma proteins as candidate blood-based biomarkers for malignant gliomas. Cancer Invest. 2014;32(8):423-9.

6. Xiong J, Zhou L, Yang M, Lim Y, Zhu YH, Fu DL, et al. ProBDNF and its receptors are upregulated in glioma and inhibit the growth of glioma cells in vitro. Neuro-Oncology. 2013;15(8):990-1007.

7. Ilhan-Mutlu A, Wagner L, Widhalm G, Wöhrer A, Bartsch S, Czech T, et al. Exploratory investigation of eight circulating plasma markers in brain tumor patients. Neurosurgical Review. 2013;36(1):45-55.

8. Chiaretti A, Aloe L, Antonelli A, Ruggiero A, Piastra M, Riccardi R, et al. Neurotrophic factor expression in childhood low-grade astrocytomas and ependymomas. Childs Nerv Syst. 2004;20(6):412-9.
